# Adaption of the temporal correlation coefficient calculation for temporal networks (applied to a real-world pig trade network)

**DOI:** 10.1186/s40064-016-1811-7

**Published:** 2016-02-24

**Authors:** Kathrin Büttner, Jennifer Salau, Joachim Krieter

**Affiliations:** Institute of Animal Breeding and Husbandry, Christian-Albrechts-University, Olshausenstr. 40, 24098 Kiel, Germany

**Keywords:** Temporal network, Temporal correlation coefficient, Topological overlap, Pig trade network

## Abstract

The average topological overlap of two graphs of two consecutive time steps measures the amount of changes in the edge configuration between the two snapshots. This value has to be zero if the edge configuration changes completely and one if the two consecutive graphs are identical. Current methods depend on the number of nodes in the network or on the maximal number of connected nodes in the consecutive time steps. In the first case, this methodology breaks down if there are nodes with no edges. In the second case, it fails if the maximal number of active nodes is larger than the maximal number of connected nodes. In the following, an adaption of the calculation of the temporal correlation coefficient and of the topological overlap of the graph between two consecutive time steps is presented, which shows the expected behaviour mentioned above. The newly proposed adaption uses the maximal number of active nodes, i.e. the number of nodes with at least one edge, for the calculation of the topological overlap. The three methods were compared with the help of vivid example networks to reveal the differences between the proposed notations. Furthermore, these three calculation methods were applied to a real-world network of animal movements in order to detect influences of the network structure on the outcome of the different methods.

## Background

In contrast to the static situation, the time when edges are active and especially the chronological order of contacts play an important role in temporal networks. Both are essential elements for the representation of these dynamical systems (Holme and Saramäki 2012). In previous studies dealing with network analysis, the temporal information has been partly neglected by an aggregation of contacts over specific observation windows, which have been analysed separately (examples of animal trade networks are Bajardi et al. 2011; Büttner et al. 2015; Dubé et al. 2011; Nöremark et al. 2011; Rautureau et al. 2011; Vernon and Keeling 2009). Even in cases where the temporal information was available, this aggregation was performed due to the fact that the methodological framework for the analysis of temporal networks is still in its infancy (Nicosia et al. 2013; Masuda and Holme 2013). However, recently, new methods for the analysis of temporal networks have been developed or methods of the static network analysis have been adapted to temporal systems. Examples are the newly proposed parameters causal fidelity by Lentz et al. (2013) or the temporal correlation coefficient, which was derived from the local clustering coefficient of static networks (Nicosia et al. 2013; Tang et al. 2010). In the case of the temporal correlation coefficient, the novelty of the temporal network analysis and the fact that its methodologies are still under development becomes obvious. Here, Pigott and Herrera (2014) presented a possible correction for the calculation of the temporal correlation coefficient proposed by Nicosia et al. (2013). The temporal correlation coefficient (hereinafter abbreviated *C*) is a measure of the overall average probability for an edge to persist across two consecutive time steps (Nicosia et al. 2013; Tang et al. 2010). For the calculation of the temporal correlation coefficient, the average topological overlaps of the graph which measures the amount of changes in the edge configuration between two consecutive time steps are determined. The values for the average topological overlap range between zero and one, whereby zero and one indicate that the edge configuration of the two consecutive graphs is completely different or has not changed at all, respectively. Current methods depend on the number of nodes in the network (Nicosia et al. 2013), hereinafter referred to as *Method 1*, or on the maximal number of connected nodes in the consecutive time steps, hereinafter referred to as *Method 2*. *Method 1* fails to deliver the value of one for identical consecutive graphs if there are nodes with no edges (Pigott and Herrera 2014), and *Method 2* delivers values greater than one if the maximal number of nodes with at least one edge is greater than the maximal size of the greatest connected component in the two consecutive graphs. The newly proposed adaption, hereinafter referred to as *Method 3*, uses the maximal number of active nodes, i.e. the number of nodes with at least one edge, for the calculation of the topological overlap. This article provides small, comprehensible examples of graphs, where the results of the temporal correlation coefficient differ between the three methods. Additionally, using all three methods, the average topological overlaps were calculated for a real-world network describing animal movements. Influences of the network structure on the differences between methods were statistically analysed.

## Methods

In the first part of the materials and methods section, the individual calculation steps of the temporal correlation coefficient are introduced, followed by a summary of the previous proposals and the adaption presented in this article with the help of vivid example networks. In the fifth part of the materials and methods section, the convergence behaviour of the three methods is compared, followed by a real-world example of a trade network of a pork supply chain.

### Temporal correlation coefficient

**Fig. 1:**
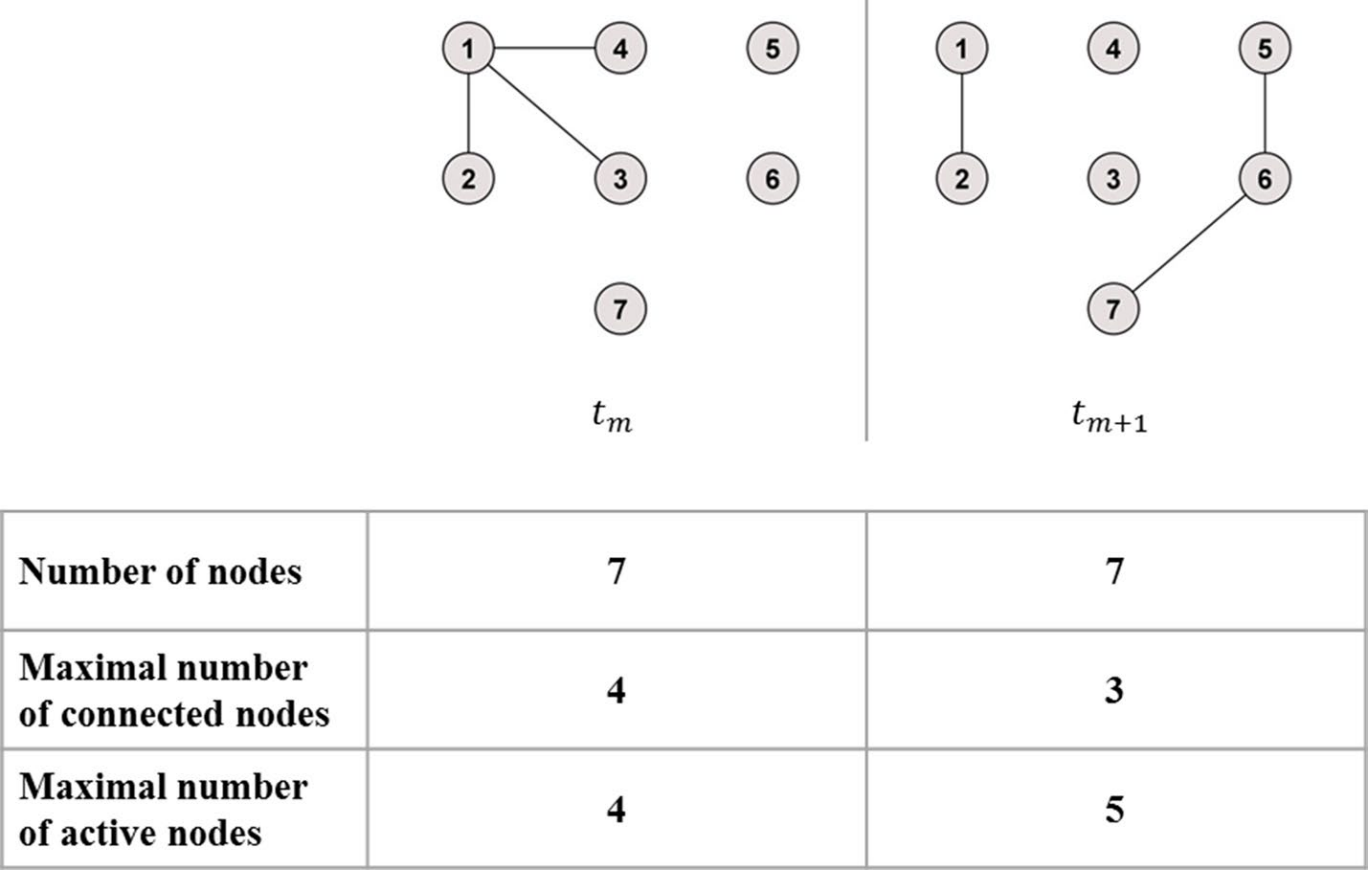
Exemplary presentation of term definitions. Illustration of the terms number of nodes, maximal number of connected nodes and maximal number of active nodes

The temporal correlation coefficient *C* is a measure of the overall average probability for an edge to persist across two consecutive time steps (Nicosia et al. 2013; Tang et al. 2010). The calculation of *C* consists of three individual calculation steps. First of all, for all nodes $i = 1, \ldots , N$, where *N* is the total number of nodes in the network *a*, and all time steps *t*_*m*_, with $m = 1, \ldots , M - 1$, where *M* is the total number of considered snapshots, the topological overlap $C_{i} \left( {t_{m} , t_{m + 1} } \right)$ in the neighbourhood of node *i* between two consecutive time steps *t*_*m*_ and *t*_*m*+1_ is calculated as

1 $$C_{i} \left( {t_{m} , t_{m + 1} } \right) = \frac{{\mathop \sum \nolimits_{j} a_{ij} \left( {t_{m} } \right)a_{ij} \left( {t_{m + 1} } \right)}}{{\sqrt {\left[ {\mathop \sum \nolimits_{j} a_{ij} (t_{m} )} \right]\left[ {\mathop \sum \nolimits_{j} a_{ij} \left( {t_{m + 1} } \right)} \right]} }},$$

where *a*_*ij*_ illustrates an entry in the unweighted adjacency matrix of the graph. Thus, summing over *a*_*ij*_ gives the interaction between *i* and every other node for two consecutive time steps *t*_*m*_ and *t*_*m*+1_. The average topological overlap of the graph *C*_*m*_ for two consecutive time steps *t*_*m*_ and *t*_*m+1*_ can then be determined. In this calculation step, the proposed correction of Pigott and Herrera (2014) and the possible adaption in the present article differ from the originally recommended method of Nicosia et al. (2013). The differences are described below and use the terms ‘maximal number of connected nodes’ and ‘maximal number of active nodes’. Hereby, the maximal number of connected nodes for the time *m* is defined as the maximum of the sizes of the largest connected components of the graph at *t*_*m*_ and *t*_*m*+1_. It is denoted by $max\left[ {N\left( {t_{m} } \right), N\left( {t_{m + 1} } \right)} \right]$. A node *i* is called “active” at time *m*, if there exists a node *j* ≠ *i* and an edge between *i* and *j* in the graph at *t*_*m*_. The maximal number of active nodes of the graph at *t*_*m*_ and *t*_*m*+1_ is denoted by $max\left[ {A\left( {t_{m} } \right), A\left( {t_{m + 1} } \right)} \right]$. For a better understanding of the given definitions, Fig. 1 illustrates the differences between number of nodes, maximal number of connected nodes and maximal number of active nodes.

In the last calculation step, the summation over all possible results for the topological overlap gives the temporal correlation coefficient of the network *C*.

2 $$C = \frac{1}{M - 1}\mathop \sum \limits_{m = 1}^{M - 1} C_{m} .$$

The values of all three calculation steps range between zero and one, with one indicating that there is a complete match of the edge configuration and zero if none the same edges is shared.

### *Method 1*: original calculation by Nicosia et al. (2013)

#### 1st step: calculation of $C_{i} \left( {t_{m} , t_{m + 1} } \right)$

Compare Eq. (1) in 2.1.

#### 2nd step: calculation of $C_{m}$

Due to better comparison between the different methods, the order of the original summation of Nicosia et al. (2013) is reversed.

3 $$C_{m} = \frac{1}{N}\mathop \sum \limits_{i = 1}^{N} C_{i} \left( {t_{m} , t_{m + 1} } \right),$$

where *N* is the number of nodes in the graph.

#### 3rd step: calculation of $C$

The summation over all possible *C*_*m*_ gives the temporal correlation coefficient *C*, compare Eq. (2) in 2.1.

According to Nicosia et al. (2013), *C*_*m*_ = 1 if and only if the two graphs of the two consecutive time steps *t*_*m*_ and *t*_*m*+1_ have exactly the same configuration of edges. *C*_*m*_ = 0 if the two graphs do not share any edges. This claim is only true if all *N* nodes considered in the calculation have at least one edge (Pigott and Herrera 2014), i.e. are active. However, this is not applicable for networks containing unconnected nodes, since for these graphs the correlation between two snapshots is underestimated.

### *Method 2*: proposed correction by Pigott and Herrera (2014)

Pigott and Herrera (2014) proposed the following correction in the second step of the calculation of the temporal correlation coefficient [see Eq. (3)]. Instead of dividing by the total number of nodes in the graph, the denominator is replaced by the maximal number of connected nodes of two consecutive time steps:

4 $$C_{m} = \frac{1}{{max\left[ {N\left( {t_{m} } \right), N\left( {t_{m + 1} } \right)} \right]}}\mathop \sum \limits_{i = 1}^{N} C_{i} \left( {t_{m} , t_{m + 1} } \right)$$

However, if the maximal number of active nodes is higher than the maximal number of connected nodes, the proposed correction leads to an overestimation of the average topological overlap (*C*_*m*_ > 1).

### *Method 3*: adaption of the calculation of the temporal correlation coefficient

If one of the two consecutive snapshots contains more than one connected component with two or more nodes, this implies $max\left[ {N\left( {t_{m} } \right), N\left( {t_{m + 1} } \right)} \right] < max\left[ {A\left( {t_{m} } \right), A\left( {t_{m + 1} } \right)} \right]$. To ensure that in this case *C*_*m*_ shows the expected behaviour for *Method 3*, $max\left[ {N\left( {t_{m} } \right), N\left( {t_{m + 1} } \right)} \right]$ is replaced by $max\left[ {A\left( {t_{m} } \right), A\left( {t_{m + 1} } \right)} \right]$. Note that this method still results in $\frac{0}{0}$ for the correlation between two networks with zero edges.

5 $$C_{m} = \frac{1}{{max\left[ {A\left( {t_{m} } \right), A\left( {t_{m + 1} } \right)} \right]}}\mathop \sum \limits_{i = 1}^{N} C_{i} \left( {t_{m} , t_{m + 1} } \right)$$

6 $$C = \frac{1}{M - 1}\mathop \sum \limits_{m = 1}^{M - 1} \left( {\frac{1}{{max\left[ {A\left( {t_{m} } \right), A\left( {t_{m + 1} } \right)} \right]}}\mathop \sum \limits_{i = 1}^{N} C_{i} \left( {t_{m} , t_{m + 1} } \right)} \right)$$

### Convergence behaviour of the temporal correlation coefficient in the three example networks

In order to reveal the convergence behaviour of the three presented methods, the last snapshot, i.e. the graph at *t*_*M*_ of the example networks, was repeatedly attached to the existing time series until the length of the series equalled 100. For all $m = 1, \ldots , M - 1$ an average topological overlap *C*_*m*_ ≤ 1 is expected. Due to the fact that the following graphs are identical to the snapshots at *t*_*M*_, all the following values for the average topological overlap equal 1. Therefore, this identical extension of the time series should show a convergence of the temporal correlation coefficient to one.

### Real-world example: pig trade network of a producer community in Northern Germany

#### Data basis

Pig movement data from a producer community in Northern Germany were recorded in an observation period from 1st June 2006 to 31st May 2009. The date of the movements, the supplier, the purchaser as well as the batch size and the type and age group of the delivered livestock were recorded. The holdings are represented by the nodes of the network and the edges illustrate the animal movements between them. In total, the data contained 4635 animal movements between 483 holdings.

#### Construction of networks with different time window lengths

In order to assess the influence of the chosen time window length on the results of the temporal correlation coefficient, time windows with increasing lengths were generated from 1 to 548 days. This implies that 1096 snapshots of the network were constructed for the time window length of 1 day, there were 548 snapshots for the time window length of 2 days, and finally there were only 2 snapshots in which the edge configuration can be compared for the time window length of 548 days. An incomplete time window remains to aggregate contacts for the last snapshot for time window lengths that are not proper divisors of 1096. Snapshots resulting from incomplete time windows were ignored in the analysis. For each time window length, the topological overlap of each two consecutive time steps were calculated using all three methods presented in “*Method 1*: original calculation by Nicosia et al. (2013)”, “*Method 2*: proposed correction by Pigott and Herrera (2014)” and “*Method 3*: Adaption of the calculation of the temporal correlation coefficient” sections. These were afterwards summarized to the temporal correlation coefficient for each time window length.

#### Statistical analysis

For the complete outcome of average topological overlap *C*_*m*_ minimal and maximal values, mean value, variance, skewness, and kurtosis were calculated within the three methods presented. The same descriptive statistics were calculated for the *C*_*m*_-differences between the methods. As *Method 2* generally showed greater *C*_*m*_ values than *Method 1* and *Method 3*, and as *Method 3* showed greater *C*_*m*_ values than *Method 1*, the differences *Method 2* − *Method 1*, *Method 2* − *Method 3*, and *Method 3* − *Method 1* were computed to ensure homogeneity in signs. In order to estimate the influence of different network properties on the differences between the three proposed methods, an analysis of variance (ANOVA) was conducted with the six main effects illustrated in Table 1. Firstly, an analysis of variance using a linear model containing only the main effects thereby neglecting the interaction effects was performed for each comparison between the three methods. In the second step, an analysis of variance was carried out using a model with the main effects and one additional interaction effect. Due to the fact that all other effects describing the interaction between two main effects showed no significant effect or cause singularities, only the interaction between Mean number and Mean first remained in the model. As a goodness-of-fit statistic, the coefficient of determination was calculated for all models. Additionally, the effect sizes $\eta^{2} = \frac{sum \;of\;squares\;due\;to\;effect}{total\;sum\;of\;squares}$ were calculated for all significant effects. The statistical analyses were carried out using the Statistics Toolbox of MATLAB (2015).

**Table 1:** Main effects used for the analysis of variance

Effect	Group boundaries	Group size
*TWL*—length of the time window chosen to analyse the development of the graph over time	TWL = 1	1096
	2 ≤ TWL ≤ 4	1184
	5 ≤ TWL ≤ 12	1106
	13 ≤ TWL 35	1110
	36 ≤ TWL ≤ 105	1080
	TWL ≥ 106	1166
*Mean number*—arithmetic mean of the number of connected components (containing more than one node) between two consecutive time steps	Mean number ≤ 4	2177
	5 ≤ Mean number ≤ 11	2324
	Mean number ≥ 12	2248
*Mean size*—arithmetic mean of the average sizes of all connected components containing more than one node between two consecutive time steps	Mean size ≤ 3	1830
	3 < Mean size ≤ 4.5	1692
	4.5 < Mean size ≤ 23	1569
	Mean size > 23	1658
*Mean edges*—arithmetic mean between the number of edges between two consecutive time steps	Mean edges ≤ 20	2327
	21 ≤ Mean edges ≤ 125	2134
	Mean edges ≥ 126	2288
*Mean first*—arithmetic mean of the sizes of the largest connected components between two consecutive time steps	Mean first ≤ 7	2235
	8 ≤ Mean first ≤ 60	2228
	Mean first ≥ 61	2286
*Mean active*-*first*—arithmetic mean of the differences between active nodes and the size of the largest network component between two consecutive time steps	Mean active-first ≤ 8	2262
	9 ≤ Mean active-first ≤ 35	2223
	Mean active-first ≥ 36	2264

## Results

### Comparison between the different methods based on vivid example networks

In the following, some general network examples are illustrated to reveal the differences between the three methods described above. For the example networks presented in Pigott and Herrera (2014), no differences between *Method 2* and *Method 3* could be obtained. Therefore, new example networks are presented in this article to identify the issues with the previous proposed formulas.

#### Time series without isolated nodes and identical unconnected components of equal size

**Fig. 2:**
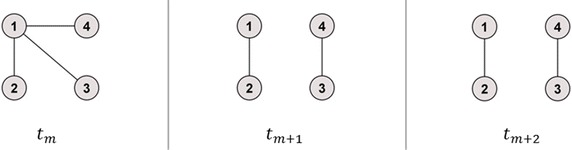
Example network 1. Connected graph becomes unconnected graph with two network components of identical size, no isolated nodes

Figure 2 illustrates the first example which shows a time series without isolated nodes and identical unconnected components of equal size. In Table 2, the single calculation steps for the temporal correlation coefficient *C* are presented depending on the different methods. For this first example, *Method 1* and *Method 3* had the same results, whereas for *Method 2* in the two snapshots *t*_*m*+1_ and *t*_*m*+2_ values above one could be obtained which exceeds the predefined upper limit for the topological overlap *C*_*m*_ as well as for the temporal correlation coefficient *C*.

**Table 2:** Calculation of the temporal correlation coefficient *C* for time series without isolated nodes and identical unconnected graphs of equal size

Snapshots	1st calculation step	2nd calculation step	3rd calculation step
$t_{m} , t_{m + 1}$	$C_{i = 1} \left( {t_{m} , t_{m + 1} } \right) = \frac{1}{\sqrt 3 }$	*Method 1*: $C_{m} = \frac{1}{N}\mathop \sum \nolimits_{i = 1}^{N} C_{i} \left( {t_{m} , t_{m + 1} } \right) \approx 0.39$ *Method 2*: $C_{m} = \frac{1}{{\text{max} \left[ {N\left( {t_{m} } \right), N\left( {t_{m + 1} } \right)} \right]}}\mathop \sum \nolimits_{i = 1}^{N} C_{i} \left( {t_{m} , t_{m + 1} } \right) \approx 0.39$ *Method 3*: $C_{m} = \frac{1}{{\text{max} \left[ {A\left( {t_{m} } \right), A\left( {t_{m + 1} } \right)} \right]}}\mathop \sum \nolimits_{i = 1}^{N} C_{i} \left( {t_{m} , t_{m + 1} } \right) \approx 0.39$	*Method 1*: $C = \frac{1}{M - 1}\mathop \sum \nolimits_{m}^{M - 1} C_{m} \approx 0.70$ *Method 2*: $C = \frac{1}{M - 1}\mathop \sum \nolimits_{m}^{M - 1} C_{m} \approx 1.20$ *Method 3*: $C = \frac{1}{M - 1}\mathop \sum \nolimits_{m}^{M - 1} C_{m} \approx 0.70$
	$$C_{i = 2} \left( {t_{m} , t_{m + 1} } \right) = 1$$		
	$$C_{i = 3} \left( {t_{m} , t_{m + 1} } \right) = 0$$		
	$$C_{i = 4} \left( {t_{m} , t_{m + 1} } \right) = 0$$		
$t_{m + 1} , t_{m + 2}$	$C_{i = 1} \left( {t_{m + 1} , t_{m + 2} } \right) = 1$	*Method 1*: $C_{m + 1} = \frac{1}{N}\mathop \sum \nolimits_{i = 1}^{N} C_{i} \left( {t_{m + 1} , t_{m + 2} } \right) = 1$ *Method 2*: $C_{m + 1} = \frac{1}{{\text{max} \left[ {N\left( {t_{m + 1} } \right), N\left( {t_{m + 2} } \right)} \right]}}\mathop \sum \nolimits_{i = 1}^{N} C_{i} \left( {t_{m + 1} , t_{m + 2} } \right) = 2$ *Method 3*: $C_{m + 1} = \frac{1}{{\text{max} \left[ {A\left( {t_{m + 1} } \right), A\left( {t_{m + 2} } \right)} \right]}}\mathop \sum \nolimits_{i = 1}^{N} C_{i} \left( {t_{m + 1} , t_{m + 2} } \right) = 1$	
	$$C_{i = 2} \left( {t_{m + 1} , t_{m + 2} } \right) = 1$$		
	$$C_{i = 3} \left( {t_{m + 1} , t_{m + 2} } \right) = 1$$		
	$$C_{i = 4} \left( {t_{m + 1} , t_{m + 2} } \right) = 1$$		

#### Time series with identical unconnected components of equal size and isolated node

**Fig. 3:**
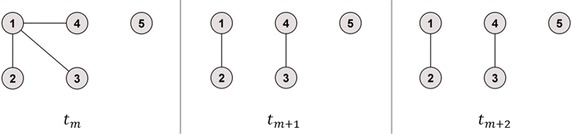
Example network 2. Unconnected graph, one network component with more than one node, one isolated node. After the first time step, the largest network component splits into two network components of identical size and one isolated node

The second example can be seen in Fig. 3, which contains time series with identical unconnected components of equal size and one isolated node. The single calculation steps for the temporal correlation coefficient are illustrated in Table 3. Compared to the first example, *Method 2* showed again values above one in the second and third calculation step. In contrast, *Method 1* revealed for the two identical snapshots *t*_*m*+1_ and *t*_*m*+2_ a value lower than one which is a clear underestimation of the real topological overlap *C*_*m*_. Only *Method 3* showed the expected behaviour of the second and the third calculation step.

**Table 3:** Calculation of the temporal correlation coefficient *C* for time series with identical unconnected components of equal size and isolated node

Snapshots	1st calculation step	2nd calculation step	3rd calculation step
$t_{m} , t_{m + 1}$	$C_{i = 1} \left( {t_{m} , t_{m + 1} } \right) = \frac{1}{\sqrt 3 }$	*Method 1*: $C_{m} = \frac{1}{N}\mathop \sum \nolimits_{i = 1}^{N} C_{i} \left( {t_{m} , t_{m + 1} } \right) \approx 0.32$ *Method 2*: $C_{m} = \frac{1}{{\text{max} \left[ {N\left( {t_{m} } \right), N\left( {t_{m + 1} } \right)} \right]}}\mathop \sum \nolimits_{i = 1}^{N} C_{i} \left( {t_{m} , t_{m + 1} } \right) \approx 0.39$ *Method 3*: $C_{m} = \frac{1}{{\text{max} \left[ {A\left( {t_{m} } \right), A\left( {t_{m + 1} } \right)} \right]}}\mathop \sum \nolimits_{i = 1}^{N} C_{i} \left( {t_{m} , t_{m + 1} } \right) \approx 0.39$	*Method 1*: $C = \frac{1}{M - 1}\mathop \sum \nolimits_{m}^{M - 1} C_{m} \approx 0.56$ *Method 2*: $C = \frac{1}{M - 1}\mathop \sum \nolimits_{m}^{M - 1} C_{m} \approx 1.20$ *Method 3*: $C = \frac{1}{M - 1}\mathop \sum \nolimits_{m}^{M - 1} C_{m} \approx 0.70$
	$$C_{i = 2} \left( {t_{m} , t_{m + 1} } \right) = 1$$		
	$$C_{i = 3} \left( {t_{m} , t_{m + 1} } \right) = 0$$		
	$$C_{i = 4} \left( {t_{m} , t_{m + 1} } \right) = 0$$		
	$$C_{i = 5} \left( {t_{m} , t_{m + 1} } \right) = 0$$		
$t_{m + 1} , t_{m + 2}$	$C_{i = 1} \left( {t_{m + 1} , t_{m + 2} } \right) = 1$	*Method 1*: $C_{m + 1} = \frac{1}{N}\mathop \sum \nolimits_{i = 1}^{N} C_{i} \left( {t_{m + 1} , t_{m + 2} } \right) = 0.80$ *Method 2*: $C_{m + 1} = \frac{1}{{\text{max} \left[ {N\left( {t_{m + 1} } \right), N\left( {t_{m + 2} } \right)} \right]}}\mathop \sum \nolimits_{i = 1}^{N} C_{i} \left( {t_{m + 1} , t_{m + 2} } \right) = 2$ *Method 3*: $C_{m + 1} = \frac{1}{{\text{max} \left[ {A\left( {t_{m + 1} } \right), A\left( {t_{m + 2} } \right)} \right]}}\mathop \sum \nolimits_{i = 1}^{N} C_{i} \left( {t_{m + 1} , t_{m + 2} } \right) = 1$	
	$$C_{i = 2} \left( {t_{m + 1} , t_{m + 2} } \right) = 1$$		
	$$C_{i = 3} \left( {t_{m + 1} , t_{m + 2} } \right) = 1$$		
	$$C_{i = 4} \left( {t_{m + 1} , t_{m + 2} } \right) = 1$$		
	$$C_{i = 5} \left( {t_{m + 1} , t_{m + 2} } \right) = 0$$		

#### Time series with identical unconnected components of different sizes and isolated nodes

**Fig. 4:**
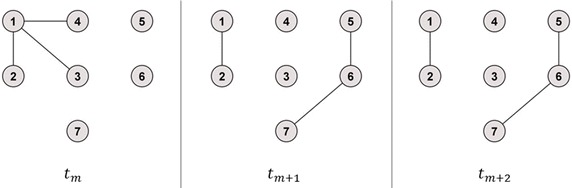
Example network 3. Unconnected graph, one network component with more than one node, one isolated node. After the first time step, two network components are formed with different sizes, two isolated nodes

Figure 4 illustrates the third example which contains time series with identical unconnected components of different sizes including isolated nodes. Table 4 presents the single calculation steps for the temporal correlation coefficient *C* for this example. Similar to the second example in Fig. 3, *Method 2* leads to an overestimation, *Method 1* leads to an underestimation and *Method 3* showed the expected behaviour of the temporal correlation coefficient regarding the two identical snapshots *t*_*m*+1_ and *t*_*m*+2_.

**Table 4:** Calculation of the temporal correlation coefficient *C* for time series with identical unconnected components of different sizes and isolated nodes

Snapshots	1st calculation step	2nd calculation step	3rd calculation step
$t_{m} , t_{m + 1}$	$C_{i = 1} \left( {t_{m} , t_{m + 1} } \right) = \frac{1}{\sqrt 3 }$	*Method 1*: $C_{m} = \frac{1}{N}\mathop \sum \nolimits_{i = 1}^{N} C_{i} \left( {t_{m} , t_{m + 1} } \right) \approx 0.23$ *Method 2*: $C_{m} = \frac{1}{{\text{max} \left[ {N\left( {t_{m} } \right), N\left( {t_{m + 1} } \right)} \right]}}\mathop \sum \nolimits_{i = 1}^{N} C_{i} \left( {t_{m} , t_{m + 1} } \right) \approx 0.39$ *Method 3*: $C_{m} = \frac{1}{{\text{max} \left[ {A\left( {t_{m} } \right), A\left( {t_{m + 1} } \right)} \right]}}\mathop \sum \nolimits_{i = 1}^{N} C_{i} \left( {t_{m} , t_{m + 1} } \right) \approx 0.32$	*Method 1*: $C = \frac{1}{M - 1}\mathop \sum \nolimits_{m}^{M - 1} C_{m} \approx 0.47$ *Method 2*: $C = \frac{1}{M - 1}\mathop \sum \nolimits_{m}^{M - 1} C_{m} \approx 1.03$ *Method 3*: $C = \frac{1}{M - 1}\mathop \sum \nolimits_{m}^{M - 1} C_{m} \approx 0.66$
	$$C_{i = 2} \left( {t_{m} , t_{m + 1} } \right) = 1$$		
	$$C_{i = 3} \left( {t_{m} , t_{m + 1} } \right) = 0$$		
	$$C_{i = 4} \left( {t_{m} , t_{m + 1} } \right) = 0$$		
	$$C_{i = 5} \left( {t_{m} , t_{m + 1} } \right) = 0$$		
	$$C_{i = 6} \left( {t_{m} , t_{m + 1} } \right) = 0$$		
	$$C_{i = 7} \left( {t_{m} , t_{m + 1} } \right) = 0$$		
$t_{m + 1} , t_{m + 2}$	$C_{i = 1} \left( {t_{m + 1} , t_{m + 2} } \right) = 1$	*Method 1*: $C_{m + 1} = \frac{1}{N}\mathop \sum \nolimits_{i = 1}^{N} C_{i} \left( {t_{m + 1} , t_{m + 2} } \right) \approx 0.71$ *Method 2*: $C_{m + 1} = \frac{1}{{\text{max} \left[ {N\left( {t_{m + 1} } \right), N\left( {t_{m + 2} } \right)} \right]}}\mathop \sum \nolimits_{i = 1}^{N} C_{i} \left( {t_{m + 1} , t_{m + 2} } \right) \approx 1.67$ *Method 3*: $C_{m + 1} = \frac{1}{{\text{max} \left[ {A\left( {t_{m + 1} } \right), A\left( {t_{m + 2} } \right)} \right]}}\mathop \sum \nolimits_{i = 1}^{N} C_{i} \left( {t_{m + 1} , t_{m + 2} } \right) = 1$	
	$$C_{i = 2} \left( {t_{m + 1} , t_{m + 2} } \right) = 1$$		
	$$C_{i = 3} \left( {t_{m + 1} , t_{m + 2} } \right) = 0$$		
	$$C_{i = 4} \left( {t_{m + 1} , t_{m + 2} } \right) = 0$$		
	$$C_{i = 5} \left( {t_{m + 1} , t_{m + 2} } \right) = 1$$		
	$$C_{i = 6} \left( {t_{m + 1} , t_{m + 2} } \right) = 1$$		
	$$C_{i = 7} \left( {t_{m + 1} , t_{m + 2} } \right) = 1$$		

### Convergence behaviour of the temporal correlation coefficient in the three example networks

**Fig. 5:**
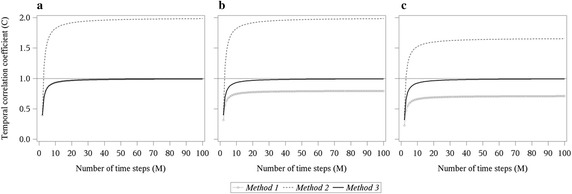
Convergence behaviour of the temporal correlation coefficient. Illustrated for the three methods described depending on the increasing number of identical time steps added to the series of the example networks of Figs. 2a, 3b, and 4c

In comparison between the three described methods, Fig. 5 shows the convergence behaviour of the temporal correlation coefficient for the example networks of Figs. 2, 3 and 4 depending on the increasing number of added identical snapshots.

For the example network of Fig. 2, *Method 1* showed the same results as the newly proposed *Method 3*, since the maximal number of active nodes equalled the maximal number of all nodes in the network. Therefore, only differences for the example networks of Figs. 3 and 4 between *Method 1* and *Method 3* could be revealed. Here, the temporal correlation coefficient converged towards the fraction of active nodes in the added identical snapshots (Pigott and Herrera 2014), which is 0.8 or 0.71, respectively, with regard to the example networks of Figs. 3 and 4.

For all three example networks, *Method 2* showed values larger than one for *M* ≥ 3. *Method 3* shows in all three example networks a convergence towards 1, which corresponds to the expected behaviour of the temporal correlation coefficient.

### Estimates of the distortions between methods

#### Averaged estimate errors for the topological overlap

For $k = 1, \ldots , 3$ the abbreviations $C_{m}^{k}$ and *C*^*k*^ denote the average topological overlap *C*_*m*_ for *t*_*m*_ and *t*_*m*+1_ and the temporal correlation coefficient *C* obtained from *Method k*, respectively. Let $m \in \left\{ {1, \ldots , M - 1} \right\}$. Then, the ratios in average topological overlaps for the time steps *t*_*m*_ and *t*_*m*+1_ between *Method 1* and *Method 2*, respectively, *Method 3* calculate to:

7 $$\frac{{C_{m}^{1} }}{{C_{m}^{2} }} = \frac{{\text{max} \left[ {N\left( {t_{m} } \right),N\left( {t_{m + 1} } \right)} \right] }}{N} \quad {\text{and}} \quad \frac{{C_{m}^{1} }}{{C_{m}^{3} }} = \frac{{\text{max} \left[ {A\left( {t_{m} } \right),A\left( {t_{m + 1} } \right)} \right] }}{N}.$$

Averaged over all time steps we get

8 $$\frac{1}{M - 1}\mathop \sum \limits_{m = 1}^{M - 1} \left( {\frac{{\text{max} \left[ {N\left( {t_{m} } \right),N\left( {t_{m + 1} } \right)} \right] }}{N}} \right) = \frac{{\mathop {\text{mean}}\nolimits_{m \le M - 1} \left( {\text{max} \left[ {N\left( {t_{m} } \right),N\left( {t_{m + 1} } \right)} \right]} \right)}}{N}.$$

#### Lower and upper boundaries for estimate errors in temporal correlation coefficients

A little more effort needs to be made to estimate the distortions between the temporal correlation coefficients. An upper boundary for the quotient $\frac{{C^{1} }}{{C^{2} }}$ was calculated as follows:

$$\begin{aligned} \frac{{C^{1} }}{{C^{2} }} & = \frac{{\frac{1}{N}\mathop \sum \nolimits_{m = 1}^{M - 1} \mathop \sum \nolimits_{i = 1}^{N} C_{i} \left( {t_{m} ,t_{m + 1} } \right)}}{{\mathop \sum \nolimits_{m = 1}^{M - 1} \frac{1}{{\text{max} \left[ {N\left( {t_{m} } \right),N\left( {t_{m + 1} } \right)} \right]}}\mathop \sum \nolimits_{i = 1}^{N} C_{i} \left( {t_{m} ,t_{m + 1} } \right)}} \\ & \le \frac{{\frac{1}{N}\mathop \sum \nolimits_{m = 1}^{M - 1} N\overbrace {{\mathop {\text{max} }\nolimits_{i \le N;m \le M - 1} \left( {C_{i} \left( {t_{m} ,t_{m + 1} } \right)} \right)}}^{ \le 1}}}{{\mathop \sum \nolimits_{m = 1}^{M - 1} \frac{1}{{\text{max} \left[ {N\left( {t_{m} } \right),N\left( {t_{m + 1} } \right)} \right]}}N\mathop {\text{min} }\nolimits_{i \le N;m \le M - 1} \left( {C_{i} \left( {t_{m} ,t_{m + 1} } \right)} \right)}} \\ & \le \frac{M - 1}{{\left( {M - 1} \right)\frac{1}{{\mathop {\text{max} }\nolimits_{m \le M} \left( {N\left( {t_{m} } \right)} \right)}}N\mathop {\text{min} }\nolimits_{i \le N;m \le M - 1} \left( {C_{i} \left( {t_{m} ,t_{m + 1} } \right)} \right)}} \\ & = \frac{{\mathop {\text{max} }\nolimits_{m \le M} \left( {N\left( {t_{m} } \right)} \right)}}{{N\mathop {\text{min} }\nolimits_{i \le N;m \le M - 1} \left( {C_{i} \left( {t_{m} ,t_{m + 1} } \right)} \right)}} \\ \end{aligned}$$

Additionally, a lower boundary could be determined:

$$\begin{aligned} \frac{{C^{1} }}{{C^{2} }} & = \frac{{\frac{1}{N}\mathop \sum \nolimits_{m = 1}^{M - 1} \mathop \sum \nolimits_{i = 1}^{N} C_{i} \left( {t_{m} ,t_{m + 1} } \right)}}{{\mathop \sum \nolimits_{m = 1}^{M - 1} \frac{1}{{\text{max} \left[ {N\left( {t_{m} } \right),N\left( {t_{m + 1} } \right)} \right]}}\mathop \sum \nolimits_{i = 1}^{N} C_{i} \left( {t_{m} ,t_{m + 1} } \right)}} \\ & \ge \frac{{\frac{1}{N}\mathop \sum \nolimits_{m = 1}^{M - 1} N\mathop {\text{min} }\nolimits_{i \le N;m \le M - 1} \left( {C_{i} \left( {t_{m} ,t_{m + 1} } \right)} \right)}}{{\mathop \sum \nolimits_{m = 1}^{M - 1} \frac{1}{{\text{max} \left[ {N\left( {t_{m} } \right),N\left( {t_{m + 1} } \right)} \right]}}N\mathop {\underbrace {{\mathop {\text{max} }\nolimits_{i \le N;m \le M - 1} \left( {C_{i} \left( {t_{m} ,t_{m + 1} } \right)} \right)}}_{ \le 1}}\nolimits_{{}} }} \\ & \ge \frac{{\left( {M - 1} \right)\mathop {\text{min} }\nolimits_{i \le N;m \le M - 1} \left( {C_{i} \left( {t_{m} ,t_{m + 1} } \right)} \right)}}{{\left( {M - 1} \right)\frac{1}{{\mathop {\text{min} }\nolimits_{m \le M - 1} \left( {\text{max} \left[ {N\left( {t_{m} } \right),N\left( {t_{m + 1} } \right)} \right]} \right)}}N}} \\ & = \frac{{\mathop {\text{min} }\nolimits_{m \le M - 1} \left( {\text{max} \left[ {N\left( {t_{m} } \right),N\left( {t_{m + 1} } \right)} \right]} \right)\mathop {\text{min} }\nolimits_{i \le N;m \le M - 1} \left( {C_{i} \left( {t_{m} ,t_{m + 1} } \right)} \right)}}{N} \\ \end{aligned}$$

For the sake of readability, the global minimum of all topological overlap values is abbreviated to min*C* = min_*i*≤*N*;*m*≤*M*−1_(*C*_*i*_(*t*_*m*_, *t*_*m*+1_)). Using this denotation the following inequalities hold:

9 $$\frac{{\mathop {\text{min} }\nolimits_{m \le M - 1} \left( {\text{max} \left[ {N\left( {t_{m} } \right),N\left( {t_{m + 1} } \right)} \right]} \right)\hbox{min}C}}{N} \le \frac{{C^{1} }}{{C^{2} }} \le \frac{{\mathop {\text{max} }\nolimits_{m \le M} \left( {N\left( {t_{m} } \right)} \right)}}{N\;\hbox{min} C}.$$

Similarly we obtained

10 $$\frac{{\mathop {\text{min} }\nolimits_{m \le M - 1} \left( {\text{max} \left[ {A\left( {t_{m} } \right),A\left( {t_{m + 1} } \right)} \right]} \right)\hbox{min} C}}{N} \le \frac{{C^{1} }}{{C^{3} }} \le \frac{{\mathop {\text{max} }\nolimits_{m \le M} \left( {A\left( {t_{m} } \right)} \right)}}{N\;\hbox{min}C},$$

and

11 $$\frac{{\mathop {\text{min} }\nolimits_{m \le M - 1} \left( {\text{max} \left[ {A\left( {t_{m} } \right),A\left( {t_{m + 1} } \right)} \right]} \right)\hbox{min}C}}{{\mathop {\text{max} }\nolimits_{m \le M} \left( {N\left( {t_{m} } \right)} \right)}} \le \frac{{C^{2} }}{{C^{3} }} \le \frac{{\mathop {\text{max} }\nolimits_{m \le M} \left( {A\left( {t_{m} } \right)} \right)}}{{\mathop {\text{min} }\nolimits_{m \le M - 1} \left( {\text{max} \left[ {N\left( {t_{m} } \right),N\left( {t_{m + 1} } \right)} \right]} \right)\hbox{min} C}}.$$

### Real-world network: trade network of a pork supply chain

#### Descriptive statistics

**Fig. 6:**
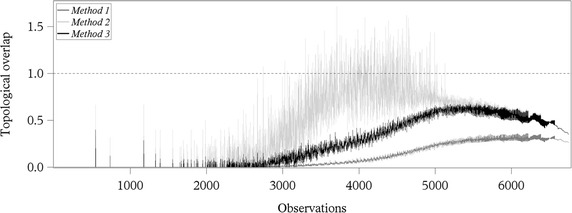
Topological overlap values. Illustrated for the three different methods calculated for the pork supply chain of a producer community in Northern Germany

Figure 6 shows the topological overlap values for each observation illustrated for the three different methods. In the arrangement of observations along the x-axis, the values determined from comparisons between snapshots with time window length 1 are displayed left. Topological overlap values calculated from comparing snapshots based on increasing time window length follow to the right. The values obtained from *Method 1* were smallest and also showed a smaller variation compared to *Method 2* and *Method 3*. These findings are confirmed by the descriptive statistics presented in Table 5.

**Table 5:** Descriptive statistics of the topological overlap values for the three different methods

	*Method 1*	*Method 2*	*Method 3*
N	6749	6749	6749
Min	0	0	0
Max	0.36	1.72	0.69
Mean	0.10	0.39	0.24
Variance	0.02	0.13	0.06
Skewness	0.76	0.36	0.40
Kurtosis	1.85	2.08	1.51

For time window lengths above 1 day (corresponding to observations number 1097 and higher), the values for the topological overlap obtained from *Method 2* and *Method 3* showed increasing behaviour up to a time window length of 53 days, which corresponds to observation number 4900 (Fig. 6). For larger time window lengths, the topological overlap values decreased again. In contrast, the values obtained from *Method 1* increased until approximately observation 6200. For both *Method 1* and *Method 3*, rising variation could be observed until observation 4900 in Fig. 6. In contrast to this, the variation of *Method 2* was reduced from that moment. Additionally, the results obtained from *Method 1* and *Method 3* remained in [0, 1] defined for the topological overlap, whereas the results calculated with *Method 2* exceeded the predefined upper limit of this parameter.

**Fig. 7:**
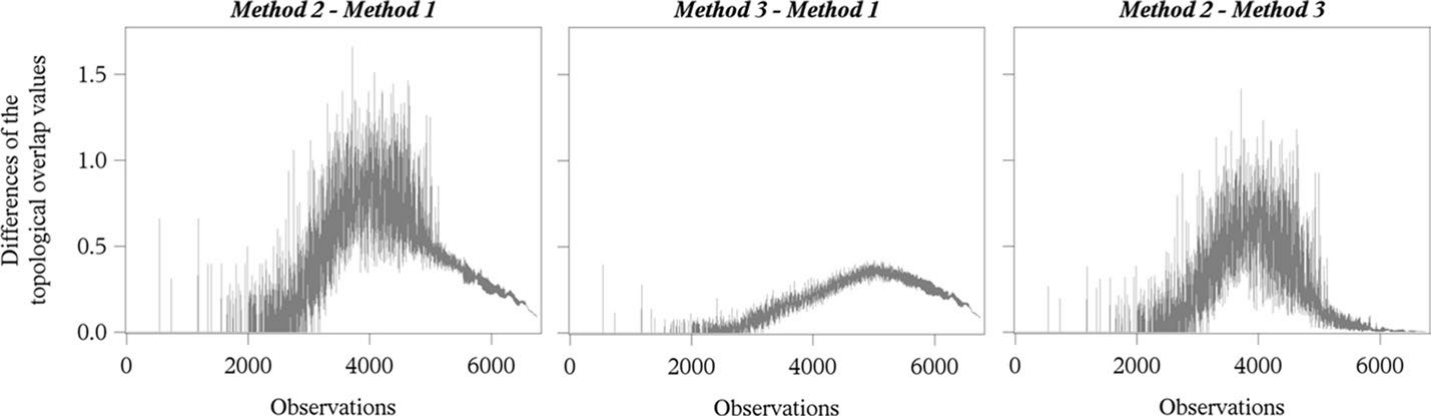
Differences of the topological overlap values for the three different methods

Figure 7 shows the differences of the topological overlap values for pairs of methods. It becomes obvious that the smallest differences could be obtained for the comparison of *Method 3* with *Method 1*, whereas the differences between *Method 2* and *Method 1* or *Method3*, respectively, showed the highest variation, which is due to the high variation in the results of the topological overlap of *Method 2* (see Fig. 6). The detailed descriptive statistics of the differences are illustrated in Table 6. It has to be noticed that the differences between *Method 2* and *Method 1* as well as between *Method 2* and *Method 3* ranged between 0 and 1.5 with their highest values around observation 4500 (time window length 36), whereas all differences between *Method 3* and *Method 1* were smaller than 1, and the largest differences could be found here approximately at observation 5200 (time window length 71).

**Table 6:** Descriptive statistics of the differences between the topological overlap values of the three methods

	*Method 2* − *Method 1*	*Method 3* − *Method 1*	*Method 2* − *Method 3*
N	6749	6749	6749
Min	0	0	0
Max	1.66	0.42	1.42
Mean	0.29	0.14	0.16
Variance	0.10	0.02	0.06
Skewness	0.97	0.36	1.65
Kurtosis	3.22	1.60	4.89

#### Analysis of variance

As the additional interaction effect between Mean number and Mean first (see Table 1) has no influence on the models’ coefficients of determination, the results are restricted to the models including only linear effects.

##### Differences of the topological overlap between *Method 2* and *Method 1*

The results of the analysis of variance using a linear model showed that all six main effects had a significant influence on the differences between the topological overlap values of *Method 2* and *Method 1* (p < 0.05). The model explained 82.4 % of the total variance (coefficient of determination). For the single main effects, most of the variance was explained by the time window length (effect size = 0.053), followed by the mean of the differences between active nodes and the size of the largest network component between two consecutive time steps (Mean active-first, see Table 1; effect size = 0.017) and the mean of the sizes of the largest network components between two consecutive time steps (Mean first, see Table 1; effect size = 0.016).

##### Differences of the topological overlap between *Method 3* and *Method 1*

The results of the analysis of variance using a linear model showed that all six main effects had a significant influence on the difference between the topological overlap values of *Method 3* and *Method 1* (p < 0.05). The model explained 91.7 % of the total variance. For the single main effects, most of the variance was explained by the time window length (effect size = 0.039), followed by the arithmetic mean of the average sizes of all connected components containing more than one node (Mean size, see Table 1; effect size = 0.004) and Mean active-first (effect size = 0.004).

##### Differences of the topological overlap between *Method 2* and *Method 3*

The results of the analysis of variance using a linear model showed that all six main effects had a significant influence on the difference between the topological overlap values of *Method 2* and *Method 3* (p < 0.001). The model explained 77.9 % of the total variance. For the single main effects, most of the variance was explained by the time window length (effect size = 0.044), followed by Mean size (see Table 1; effect size = 0.038) and Mean active-first (see Table 1; effect size = 0.020).

## Discussion

The intention of this article was to eliminate uncertainties for the calculation of the topological overlap and the temporal correlation coefficient proposed by Nicosia et al. (2013) and its extension proposed by Pigott and Herrera (2014) and to give clear definitions of the network parameters used for their calculations. Therefore, we proposed comprehensive example networks which included more possible network configurations (e.g. the network contained more than one network component with more than one node) than the example networks included in Pigott and Herrera (2014). Additionally, we introduced the results of the topological overlap of a real-world network of animal movements, which revealed the problems of the previous formulas. The influences of the network structure on the outcome of the different methods were analysed with the help of this trade network.

### Expected behaviour of the topological overlap and the temporal correlation coefficient

Since the topological overlap represents the probability for edges to persist across two consecutive time steps and the temporal correlation coefficient is the average over all topological overlap values, both should range between 0 and 1. Thus, values above the upper limit of one cannot be interpreted. The present article shows that only the results obtained from *Method 1* and *Method 3* remained in [0,1], whereas the results calculated with *Method 2* exceeded the predefined upper limit of this range. This becomes obvious for the small example networks as well as for the real-world trade network. Additionally, the fact that values greater than one were determined for *Method 2* suggests that also the values in the expected range overestimated the real topological overlap and, therefore, led to invalid results. Similarly, *Method 1* converged towards a value smaller than one in Fig. 5b, c, where the maximal number of connected nodes did not equal the maximal number of active nodes. Here, the possible topological overlap and the temporal correlation coefficient were underestimated. A detailed discussion of the estimates of the distortions between the three methods is given in the following paragraph.

### Estimates of the distortions between methods

Given the presence of isolated (i.e. not active) nodes in one of the snapshots *t*_*m*_ or *t*_*m*+1_, the originally proposed *Method 1* systematically outputs a smaller topological overlap between those network snapshots than both recently proposed methods. This was e.g. illustrated in the *Calculation of C*_*m*_ associated with the example network of Fig. 4. The ratios in Eq. (7) are always smaller or equal to one and quantify the underestimation in the average topological overlap values for the time step from *t*_*m*_ to *t*_*m*+1_ obtained from *Method 1* in comparison to *Method 2* and *Method 3* for a fixed $m = 1, \ldots , M - 1$. Consequently, the right side of Eq. (8) states the averaged underestimation concerning the topological overlap caused by *Method 1* compared to *Method 2* over time. A similar estimation can be found in Pigott and Herrera (2014). Respectively, the topological overlap is averagely underestimated using *Method 1* compared to the newly proposed *Method 3* by the fraction $\frac{{\mathop {\text{mean}}\nolimits_{m \le M - 1} \left( {\text{max} \left[ {A\left( {t_{m} } \right),A\left( {t_{m + 1} } \right)} \right]} \right)}}{N} \le 1$.

If the maximal number of connected nodes $\hbox{max} \left[ {N\left( {t_{m} } \right),N\left( {t_{m + 1} } \right)} \right]$ is not equal to the maximal number of active nodes $\hbox{max} \left[ {A\left( {t_{m} } \right),A\left( {t_{m + 1} } \right)} \right]$ for a fixed $m = 1, \ldots , M - 1$, the distortion in *C*_*m*_ between *Method 2* and *Method 3* is represented by the fraction $\frac{{\text{max} \left[ {N\left( {t_{m} } \right),N\left( {t_{m + 1} } \right)} \right]}}{{\text{max} \left[ {A\left( {t_{m} } \right),A\left( {t_{m + 1} } \right)} \right]}} \ge 1$. This is underpinned by calculations for the example network of Fig. 2. Here *C*_*m*+1_ = 2 and *C*_*m*+1_ = 1 when obtained from *Method 2*, respectively, *Method 3*, whilst max [*N*(*t*_*m*+1_), *N*(*t*_*m*+2_)] = 4 and max [*A*(*t*_*m*+1_), *A*(*t*_*m*+2_)] = 2.

As the average topological overlap *C*_*m*_ has no explanatory power concerning the complete dynamic network, the distortions between methods in temporal correlation coefficient *C* should be considered in addition. Due to the double sum in the formula to calculate *C*, less transformation with equality sign is possible, but estimations are necessary. The inequalities (9)–(11) give upper and lower boundaries using characteristics of the network, as maximal and minimal values of max [*N*(*t*_*m*+1_), *N*(*t*_*m*+2_)] and max [*A*(*t*_*m*+1_), *A*(*t*_*m*+2_)] over time. They might provide a valuable tool in assessing the distortion connected to the usage of the different methods.

### Real-world network: trade network of a pork supply chain

For the pig trade network, the results of the topological overlap values showed for *Method 2* a completely different behaviour than for *Method 1* and *Method 3* (Fig. 6). For *Method 2*, the topological overlap values varied over a huge range until observation 4900. This can be explained by the variation in the differences between the maximal number of connected and the maximal number of active nodes. These differences became smaller with increasing time window length, since for larger time window length the network formed larger network components which included the majority of the nodes. Thus, the differences between the maximal number of connected and active nodes decreased, which resulted in a smaller variation.

#### Results in analysis of variance

With regard to the real-world example given by the described pig trade network, the differences of *C*_*m*_ between methods (*Method 2* − *Method 1*, *Method 2* − *Method 3*, *Method 3* − *Method 1*) were analysed with linear models containing six categorical variables chosen from the characteristics of the underlying network. The goal was to analyse the impact of the network structure on the differences in methods. As—except for the time window length—two snapshots are needed to calculate *C*_*m*_, the categorical variables are determined as the characteristics’ mean value between two consecutive snapshots. The models used successfully explained the variance in the target variables, as coefficients of determination ranged from 0.78 to 0.92. All six chosen effects were significant in all three cases, but the time window length was the strongest effect in all three considered differences and showed medium effect sizes from 0.038 to 0.055 (Cohen 1988). The remaining effects used the number and size of connected components or the total number of edges in the snapshots at *t*_*m*_ and *t*_*m*+1_. When the time windows for the aggregation of pig trade activities became longer, more edges and fewer but larger connected components are to be expected in the snapshots, but significant interaction effects between time window length and the remaining categorical variables have to be excluded in advance. The effect Mean active-first categorises the difference “size of the largest connected component − number of active nodes” averaged between the two considered snapshots. It was to be expected that its effect size was medium concerning *Method 2* − *Method 3* and only small for the other two target variables since these methods differ exactly in the terms max [*N*(*t*_*m*+1_), *N*(*t*_*m*+2_)] and max [*A*(*t*_*m*+1_), *A*(*t*_*m*+2_)].

#### General aspects

The description of temporal networks as well as the analysis of their structural characteristics is still under development (Nicosia et al. 2013). Therefore, there is still a lack of appropriate methods which help to analyse how the structure of temporal networks influences the dynamics of processes occurring on it, such as disease transmission. Furthermore, the question which characteristics of the network impact the dynamics is still not fully answered. Konschake et al. (2013) investigated the structural dynamics of a pig trade network and found that time-independent node centrality has to be treated with caution, whereas the stationary sampling of the nodes is still applicable for the network under representation. They also stated that similar results are expected for other pig trade networks since the processes in the pork supply chain are highly standardized and industrialized. A further issue, which was revealed in the present study, is the choice of an appropriate time window length. Also Clauset and Eagle (2012) stated, that the choice of the time window length effectively determines many of the statistical properties of the resulting network and that an incorrect choice may impose a strong bias on the resulting analysis and conclusion. Additionally, they could show that a time window length which displays the natural periodicity of the system should be chosen which depends on the interactions under investigation. For a pig trade network, Lentz et al. (2013) showed a periodical pattern of 180 days which represents the biological properties of pig production from farrowing to abattoir. Also Valdano et al. (2015) stated that the extent of the time window length may affect the prediction of the epidemic threshold and the spreading potential within a temporal network. Furthermore, their study confirmed the findings from other investigations that the network’s typical timescale and the temporal variability of its structure should definitely be considered for the analysis of dynamic systems. Therefore, the static aggregation of temporal networks should be treated with caution due to the fact that this approach neglects the temporal variation in the system which is of special importance for the analysis of the speed and the extent of infectious diseases (Kempe et al. 2002; Holme and Saramäki 2012; Tantipathananandh et al. 2007). To sum up, regarding the yet known dependencies and issues dealing with temporal network analysis, a measure like the temporal correlation coefficient which evaluates the consistency of the edge configuration could help to understand the structural dynamics of temporal networks.

## Conclusion

In this study, an adaption for a method to calculate the average topological overlap *C*_*m*_ between two consecutive snapshots of a dynamic network was proposed and compared to the original method and another recently proposed adaption. The methods differ in the kind of nodes used to average the changes in edge configuration. The numerical differences between the methods were demonstrated using several small and clearly arranged example networks, and analytical estimations were given as well. A pig trade network was introduced and statistically analysed as a real-world example. The newly proposed *Method 3* uses the maximal number of active nodes in two consecutive snapshots. Solely for *Method 3*, the temporal correlation coefficient shows convergence behaviour towards one and, additionally, the values for the topological overlap equals one (*C*_*m*_ = 1) in cases where consecutive snapshots are identical with regard to all given examples. Both are expected behaviours for a measure of temporal correlation between graphs.

## References

[CR1] Bajardi P, Barrat A, Natale F, Savini L, Colizza V (2011). Dynamical patterns of cattle trade movements. PLoS One.

[CR2] Büttner K, Krieter J, Traulsen I (2015). Characterization of contact structures for the spread of infectious diseases in a pork supply chain in northern germany by dynamic network analysis of yearly and monthly networks. Transbound Emerg Dis.

[CR3] Clauset A, Eagle N (2012) Persistence and periodicity in a dynamic proximity network. arXiv preprint arXiv:12117343

[CR4] Cohen J (1988). Statistical power analysis for the behavioral sciences.

[CR5] Dubé C, Ribble C, Kelton D, McNab B (2011). Estimating potential epidemic size following introduction of a long-incubation disease in scale-free connected networks of milking-cow movements in Ontario, Canada. Prev Vet Med.

[CR6] Holme P, Saramäki J (2012). Temporal networks. Phys Rep.

[CR7] Kempe D, Kleinberg J, Kumar A (2002). Connectivity and inference problems for temporal networks. J Comput Syst Sci.

[CR8] Konschake M, Lentz HHK, Conraths FJ, Hövel P, Selhorst T (2013). On the robustness of in- and out-components in a temporal network. PLoS One.

[CR9] Lentz HHK, Selhorst T, Sokolov IM (2013). Unfolding accessibility provides a macroscopic approach to temporal networks. Phys Rev Lett.

[CR10] Masuda N, Holme P (2013). Predicting and controlling infectious disease epidemics using temporal networks. F1000Prime Rep.

[CR11] MATLAB (2015). Statistics and machine learning toolbox™ user’s guide (version 2014a).

[CR12] Nicosia V, Tang J, Mascolo C, Musolesi M, Russo G, Latora V, Holme P, Saramäki J (2013). Graph metrics for temporal networks. Temporal networks.

[CR13] Nöremark M, Hakansson N, Lewerin SS, Lindberg A, Jonsson A (2011). Network analysis of cattle and pig movements in Sweden: measures relevant for disease control and risk based surveillance. Prev Vet Med.

[CR14] Pigott F, Herrera M (2014) Proposal for a correction to the temporal correlation coefficient calculation for temporal networks. arXiv preprint arXiv:14031104

[CR15] Rautureau S, Dufour B, Durand B (2011). Structural vulnerability of the French swine industry trade network to the spread of infectious diseases. Animal.

[CR16] Tang J, Scellato S, Musolesi M, Mascolo C, Latora V (2010). Small-world behavior in time-varying graphs. Phys Rev E.

[CR17] Tantipathananandh C, Berge-Wolf T, Kempe D (2007) A framework for community identification in dynamic social networks. In: Paper presented at the Proceedings of the 13th ACM SIGKDD international conference on knowledge discovery and data mining, San Jose, California, USA

[CR18] Valdano E, Ferreri L, Poletto C, Colizza V (2015). Analytical computation of the epidemic threshold on temporal networks. Phys Rev X.

[CR19] Vernon MC, Keeling MJ (2009). Representing the UK’s cattle herd as static and dynamic networks. Proc R Soc B.

